# Molecular Modeling Study on Tunnel Behavior in Different Histone Deacetylase Isoforms

**DOI:** 10.1371/journal.pone.0049327

**Published:** 2012-11-29

**Authors:** Sundarapandian Thangapandian, Shalini John, Yuno Lee, Venkatesh Arulalapperumal, Keun Woo Lee

**Affiliations:** Division of Applied Life Science (BK21 Program), Systems and Synthetic Agrobiotech Center (SSAC), Plant Molecular Biology and Biotechnology Research Center (PMBBRC), Research Institute of Natural Science (RINS), Gyeongsang National University (GNU), Gazwa-dong, Jinju, Republic of Korea.; Goethe University, Germany

## Abstract

Histone deacetylases (HDACs) have emerged as effective therapeutic targets in the treatment of various diseases including cancers as these enzymes directly involved in the epigenetic regulation of genes. However the development of isoform-selective HDAC inhibitors has been a challenge till date since all HDAC enzymes possess conserved tunnel-like active site. In this study, using molecular dynamics simulation we have analyzed the behavior of tunnels present in HDAC8, 10, and 11 enzymes of class I, II, and IV, respectively. We have identified the equivalent tunnel forming amino acids in these three isoforms and found that they are very much conserved with subtle differences to be utilized in selective inhibitor development. One amino acid, methionine of HDAC8, among six tunnel forming residues is different in isoforms of other classes (glutamic acid (E) in HDAC10 and leucine (L) in HDAC 11) based on which mutations were introduced in HDAC11, the less studied HDAC isoform, to observe the effects of this change. The HDAC8-like (L268M) mutation in the tunnel forming residues has almost maintained the deep and narrow tunnel as present in HDAC8 whereas HDAC10-like (L268E) mutation has changed the tunnel wider and shallow as observed in HDAC10. These results explained the importance of the single change in the tunnel formation in different isoforms. The observations from this study can be utilized in the development of isoform-selective HDAC inhibitors.

## Introduction

**Figure 1:**
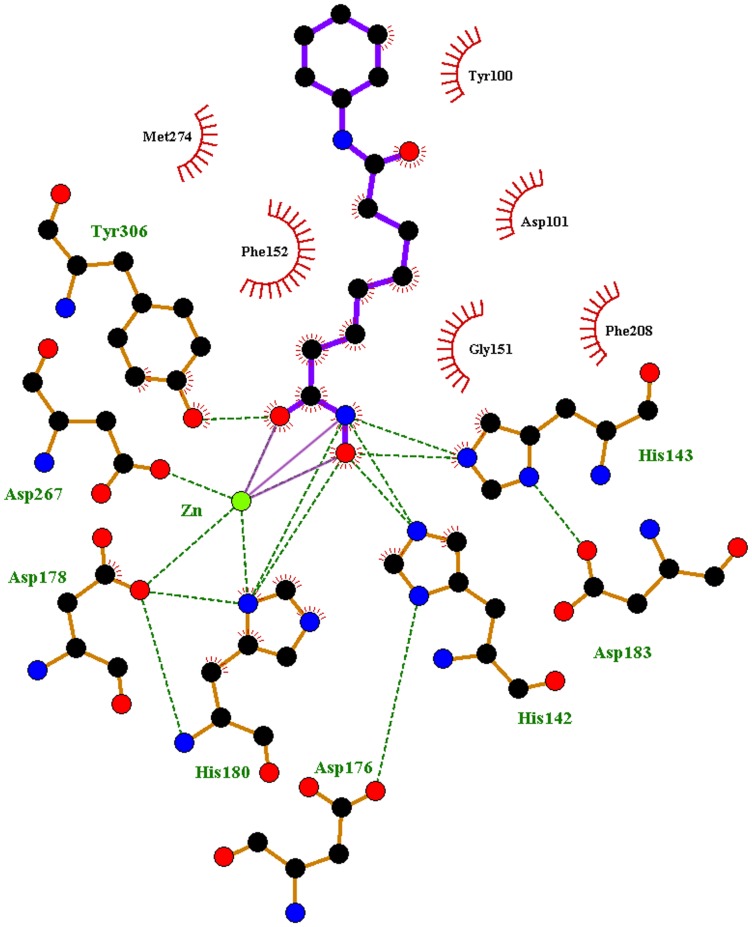
A diagrammatic representation of ligand binding at the tunnel-like active site of HDAC8. The binding of SAHA, the first FDA approved drug for HDAC inhibition, along with arrangement of charge relay system and tunnel-forming residues and their interactions with the ligand are denoted.

Chromatin that is found inside the nuclear envelope of eukaryotic cells is the combination of DNA, highly basic histones, and other proteins that form chromosomes [1]. The functions of chromatins are to package DNA into a smaller volume to fit in the cell, to strengthen the DNA to allow mitosis, meiosis, apoptosis, and to control gene expression and DNA replication [2]. The reversible change of the chemical state of ε-amino group of the lysine residues present in the N-termini of core histone proteins greatly affects the chromatin remodeling and the epigenetic regulation of genes [3]. Acetylation and deacetylation of these lysine residues catalyzed by histone acetyl transferase (HAT) and histone deacetylase (HDAC) enzymes directly influences the chromatin modification and thereby the active gene expression [4]. HDACs regulate the acetylation states of histones and several other nuclear and non-nuclear proteins such as tubulin and HSP90 [5]. Eighteen HDACs were identified in humans and were divided into two major types based on their mechanism of action and catalytic machinery (Zn^2+^ and NAD^+^) present in the active site, namely, NAD- and Zn-dependent HDAC enzymes. The NAD-dependent HDAC enzymes are also known as sirtuins. The Zn-dependent HDACs were further classified in to three classes based on domain organization and phylogenetic relationship [6]. Class I HDAC enzymes comprise 1–3 and 8 isoforms and class II HDACs include 4–7, 9, and 10 whereas class IV contains only one isoform HDAC11, which is phylogenetically close to class I than class II isoforms. Class II enzymes were further classified into two subclasses, namely, IIa (HDACs 4, 5, 7, and 9) and IIb (HDACs 6 and 10) based on domain organization and sequence homology. All NAD-dependent HDACs called sirtuins were grouped as class III HDAC enzymes [7]. HDAC6, one of the two class IIb HDACs, is unique among the entire family consisting two independent catalytic domains and a zinc finger ubiquitin-binding domain at its C-termini whereas the other class IIb isoform, HDAC10, lacks the complete second catalytic domain [8]. Class I, II and IV HDACs share sequence and structural homology within their catalytic domains but in contrast, the sirtuins do not share sequence or structural homology with other HDAC family members and catalyze the deacetylation mechanism using the oxidized form of NAD^+^ cofactor [9], [10]. The balance between acetylation and deacetylation is an important factor in regulating gene expression and thus associated to the control of cell fate. An imbalance of HAT and HDAC activity is possibly associated with cancer development [11]. HDACs have been implicated for the first time in cancer while studying acute promyelocytic leukemia [12]. Since then, HDAC silencing or inhibition has shown to have an impact on cell cycle, cell growth, chromatin decondensation, cell differentiation, apoptosis, and angiogenesis in several cancer cell types [13]. The inhibition of HDAC enzymes has proven to induce antiproliferative effects and cellular differentiation. Thus inhibition of HDACs has emerged as a promising strategy to reverse the epigenetic changes related to cancer and many other diseases [14]–[16]. Various computational methodologies using structure and ligand-based approaches were employed in designing novel HDAC inhibitors in recent times. The outcomes of these computational studies were useful and effective in designing linkerless and isoform-specific HDAC inhibitors [8], [17], [18]. The elucidation of first crystal structure of HDAC-related protein, the histone deacetylase-like protein of *Aquifex aeolicus* in the year 1999 followed by human HDAC8 and other isoforms have led to the structure-based HDAC inhibitor design. The comparison of the crystal structures of HDAC enzymes solved to date suggested that a tunnel-like active site is present in all the HDAC enzymes (Fig. 1) [19], [20]. However, the dimensions of the tunnel are different in every isoform (Fig. S1). The X-ray structures of any of class IIb and IV HDAC enzymes are not solved so far and remain untouched in terms of drug discovery from a structural perspective. The recently solved crystal structures of enzyme isoforms such as HDAC4 and HDAC7 from class IIa HDACs reported that a similar overall fold was observed compared to other HDAC structures [21], [22]. Though the overall fold is similar between different isoforms there are subtle differences in and around the catalytic tunnel-like active site which can be utilized in isoform-selective HDAC inhibitors. In terms of class I enzymes, HDAC1 and HDAC8 are the most studied enzymes but the crystal structure of HDAC1 remains unsolved whereas a set of structures are available for HDAC8 co-crystallized with different inhibitors. Except HDAC8, functional HDACs are not found as single peptides but as multimeric complexes of higher molecular weight. In addition, most of the purified HDAC enzymes are functionally inactive [23]–[25]. These characteristics of HDAC8 make it a good target for molecular modeling. Previous studies on developing isoform-selective HDAC inhibitors have concluded that exploitation of 11 Å long tunnel-like active site of different isoforms is difficult to introduce the isoform selectivity as they are very much conserved and also about the intrinsic differences in substrate selectivity in different isoforms [17], [26]–[29]. Some studies have reported that the subtle differences in the shape and charge distribution around the entrance of the tunnel can effectively be used to address the isoform selectivity. But yet this hypothesis may not be applicable to the isoforms that are conserved even at their entrance-forming residues [30].

In this study, we have compared the behavior of tunnels present in HDAC8, HDAC10 and HDAC11, which represent class I, II and IV HDACs, respectively. The homology models of HDAC10 and HDAC11 were built using homologous HDACs from same or other classes. These homology models were validated and then used in molecular dynamic (MD) simulations to observe the tunnel behavior with and without substrate or inhibitor. The tunnel behaviors including the size of the tunnel were observed in all three isoforms and compared with one another to observe the unique differences that can be utilized in designing isoform-selective HDAC inhibitors.

## Materials and Methods

### Comparison of key tunnel forming residues

All divalent metal ion dependent HDAC enzymes possess a tunnel-like active site with a Zn^2+^ ion at the bottom to process the acetylated lysine residues of histone proteins. These tunnel-like active sites were found to be very much conserved in all HDAC isoforms regardless of their classes. The primary sequences of HDAC8 from Class I, HDAC10 of class II, and HDAC11, which is the only isoform from class IV, were aligned and compared to analyze the conservation of tunnel forming residues. *Align Multiple Sequences* protocol of Accelrys Discovery studio 2.5 (DS) (Accelrys Inc., San Diego, USA) was used in aligning the primary sequences of all HDAC enzymes under study. Multiple alignment scoring matrix, gap opening, and extension penalties were set to BLOSUM, 10.0, and 0.05, respectively. The primary sequences of all three HDAC enzymes were retrieved from UniProtKB, the protein knowledgebase, database that provides comprehensive and high quality freely accessible protein sequence and functional information [31].

### Homology modeling

Homology modeling is one of the best and reliable ways to produce 3D structure of a protein for which no crystal structures were determined. As it is also known as comparative protein modeling, this process requires a crystallographically determined 3D structure of a comparatively similar (homologous) protein, which is widely called as template. Homology modeling starts with the primary sequence comparison of target and template proteins. The identification of templates and alignment of sequences are often automated utilizing various open access servers and standalone programs such as BLAST from NCBI [32] and DS from Accelrys Inc. The reliability of the homology modeled protein is completely based on the sequence alignment and thus it is the very important and limiting step in the process of homology modeling. In this study, HDAC10 and HDAC11 enzymes were built using *Build Homology Models* protocol that runs *MODELER* algorithm as implemented in DS [33]. The BLAST tool was used to identify the possible template structures from Protein Data Bank (PDB). The top ranked structures based on the identity towards the target sequence was selected as template structure in modeling target HDAC enzymes. In terms of HDAC8, the crystal structure bound with SAHA (PDB code: 1T69) was used and the missing N-terminal and a central regions in the crystal structure were built using DS. The 3D structure of HDAC10 was built using the crystal structure of HDAC4 as template. The best model was selected upon the comparison of the stereochemical quality of the models. The 3D structure of HDAC11 was built using one of the HDAC8 crystal structures as this only class IV isoform is phylogenetically close to class I HDACs than that of class II. Ten homology models were built for every target protein and the regions that were not aligned with identical equivalent parts of the templates were considered variable regions and optimized further by selecting *High Level* of *Optimization* during the modeling. The *MODELER* program is able to simultaneously incorporate structural data from one or more reference proteins. Structural features in the reference proteins are used to derive spatial restraints which, in turn, are used to generate model protein structures using conjugate gradient and simulated annealing optimization procedures [34]. The stereochemical quality of the constructed models was assessed using various structure assessment tools such as PROCHECK, WhatCheck, and PROSA-web and the manual investigation of important characteristics 35–37.

### Molecular dynamics simulations

Initial coordinates for the protein atoms were taken from the X-ray structure of HDAC8 (PDB code: 1T69) and constructed homology models of HDAC10 and HDAC11 enzymes. The protonation states of all ionizable residues were set to their normal states at pH 7. Five MD simulations were performed for systems including apoforms of HDAC8, HDAC10, HDAC11 wild type (WT), HDAC11 with mutant L268M and HDAC11 with mutant L268E. All MD simulations were performed with GROMOS96 43a1 forcefield using GROMACS 4.0.5 package running on a high performance linux cluster computer [38], [39]. During the MD simulations, all the protein atoms including divalent metal ion (Zn^2+^) were surrounded by a cubic water box of SPC3 water molecules that extended 10 Å from the protein and periodic boundary conditions were applied in all directions. The systems were neutralized with Na^+^ and Cl^−^ counter ions replacing the water molecules and energy minimization was performed using steepest descent algorithm for 10,000 steps. A 100 ps position restrained MD simulations were performed for every system followed by 5 ns production MD simulations with a time step of 2 fs at constant pressure (1 atm) and temperature (300 K). The electrostatic interactions were calculated by the particle mesh Ewald (PME) algorithm and all bonds were constrained using LINCS algorithm. A twin range cutoff was used for long-range interactions including 0.9 nm for van der Waals and 1.4 nm for electrostatic interactions. The snapshots were collected at every 1 ps and stored for further analyses. The system stability and behavior of tunnels present in every system were analyzed using tools available with GROMACS 4.0.5 and PyMol programs.

### Validation of MD simulation results

Two validation procedures based on molecular docking were employed to confirm the reliability of the simulated structures to be used in structural analyses. A set of hydroxamic acid inhibitors co-crystallized with HDAC8 and a set of known inhibitors for HDAC8, 10 and 11 were used in this study. These sets of inhibitors were docked into the respective HDAC isoform using GOLD 5.1 program. The GOLD program from Cambridge Crystallographic Data Centre, UK uses a genetic algorithm to dock the small molecules into the protein active site. The GOLD allows for a full range of flexibility of the ligands and partial flexibility of the protein. Protein coordinates from the respective HDAC isoforms obtained from MD simulations were used to define the active site. The active site was defined with a 10 Å radius around the Zn^2+^ metal ion. The ten top-scoring conformations of every ligand were saved at the end of the calculation. Early termination option was used to skip the genetic optimization calculation when any five conformations of a particular compound were predicted within an RMS deviation value of 1.5 A. The GOLD fitness score is calculated from the contributions of hydrogen bond and van der Waals interactions between the protein and ligand, intramolecular hydrogen bonds, and strains of the ligand [40].

## Results and Discussion

### Comparison of key tunnel forming residues

**Figure 2:**
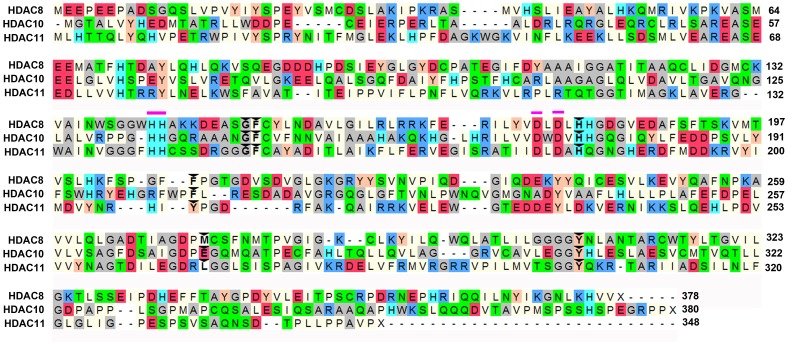
Sequence alignment of HDAC8, 10, and 11 enzymes.

The active site present in HDAC8 enzyme consists of a long and narrow tunnel leading to a cavity that contains the catalytic machinery. This tunnel-like active site accommodates the four methylene groups of the substrate, acetylated lysine, or the hydrophobic linker of inhibitor molecules. This tunnel is hydrophobic since the sidewalls of this tunnel are formed by G151, F152, H180, F208, M274, and Y306 residues of HDAC8 [19]. Multiple sequence alignment of HDAC8, 10, and 11 from class I, II and IV, respectively, has revealed the conservation of these tunnel residues. Interestingly, out of six residues that are forming the walls of tunnel, only one residue M274 of HDAC8 was different comparing with other HDAC isoforms whereas other five residues were identical or similar (Fig. 2). We found the list of equivalent tunnel forming amino acids of HDAC10 and 11 from the multiple sequence alignment with HDAC8 (Table 1). The E272 and L268 were found in HDAC10 and HDAC11 as equivalent residues to M274 that is one of the tunnel forming residues in HDAC8. There are number of inhibitors already tested for the inhibition of HDAC8 and 10. But the HDAC11 is the new member to the class IV HDAC family and not many chemical compounds were tested as HDAC11 inhibitors. In addition, the isoform specificity has been a real challenge in the area of HDAC inhibition. As an attempt to investigate the behavior of the tunnel-like active site and implicitly to find out potential isoform-selective inhibitors, we have mutated L268 of HDAC11 to methionine and glutamic acid residue in order to mimic HDAC8 and 10. These mutations were introduced to observe the structural changes of HDAC11 due to the single amino acid difference in tunnel wall. Since the crystal structures of HDAC10 and 11 are not determined yet, we have built the 3D structures of these enzymes using homology modeling methodology.

**Table 1:** Tunnel forming residues of three different isoforms HDAC8, HDAC10, and HDAC11.

Tunnel forming pair	HDAC8	HDAC10	HDAC11
Pair I	G151 – M274	G143 – E272	G151 – L268
Pair II	F208 – Y306	F205 – Y305	Y209 – Y304
Pair III	F152 – H180	F144 – H174	F152 – H183

### Homology modeling

**Figure 3:**
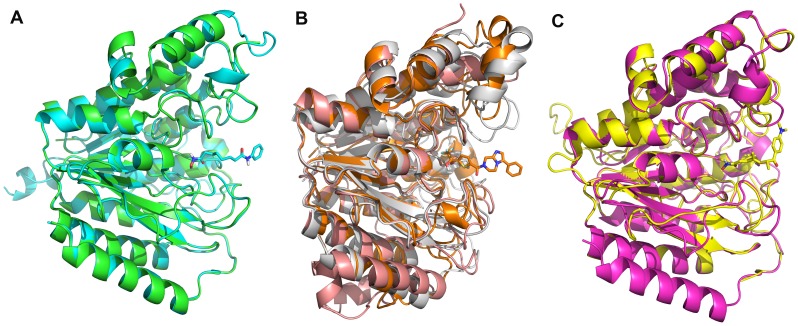
Constructed homology models of HDAC enzymes. (A) Homology model of HDAC8 (model and template in cyan and green). (B) Homology model of HDAC10 based on 2VQJ (model and template in orange and grey). (C) Homology model of HDAC11 (model and template in yellow and pink). The co-crystallized inhibitor molecules that were copied into models during homology modeling process are shown in stick form.

The crystallographic structures of human HDAC8 (class I) complexed with various hydroxamic acid inhibitors were determined at resolutions of 1.9 Å (TSA), 2.3 Å (MS-344), 2.9 Å (SAHA), and 2.2 Å (CRA-19156) (PDB codes: 1T64, 1T67, 1T69, and 1VKG, respectively). The crystal structure with the inhibitory molecule, TSA, is different compared to structures with other inhibitors by containing two molecules of TSA in two different cavities that are adjacent to each other. Because of this behavior observed in HDAC8 structure with TSA, 1T64 has not been selected as a representative structure of HDAC8 regardless of its high resolution. Another HDAC8 structure bound with inhibitory molecule, SAHA, (PDB code: 1T69) was selected to be used in this study as a representative structure for class I HDACs. This crystal structure was also selected because of the bound hydroxamic acid based inhibitor with long alkyl chain linker that resembles the lysine side chain and less number of missing residues compared to 1T67. The missing regions identified in 1T69 were built using the primary sequence of HDAC8 and 1T69 crystal structure as template employing DS (Fig. S2). As there is no 3D structure is available for HDAC10 and 11 isoforms, we built homology models using homologous proteins as templates (Text S1, Fig. S3 and S4). All the homology models (Fig. 3) were subjected to structural assessment to check their stereochemical quality based on Ramachandran statistics, which predicts the percentage of residues present in core, allowed, generally allowed, and disallowed regions (Fig. S5). The percentage of residues predicted in the disallowed regions was acceptable for both models (Table 2). The goodness factor (G factor), which represents how normal or unusual the residue's location is on the Ramachandran plot, was calculated for all the models. Analyses of bad contacts, bond lengths, bond angles, Z scores from Ramachandran plot, and ProSA predictions concluded that the models are reliable for further studies.

**Table 2:** Key geometric parameters used for validation of the HDAC10 and HDAC11 models.

HDAC Models	Template	Ramachandran plot									PROSA Z-score
		Core (%)	Allow (%)	Gener (%)	Disall (%)	Bad contacts	G-factor^a^	M/c bond lengths (%)	M/c bond angles (%)	Planar groups (%)	
HD10	HD4	89.7	7.8	1.2	1.2	20	−0.19	97.4	91.5	100	−6.31
HD11	HD8	82.3	12.4	3.7	1.7	20	−0.48	96.1	86.9	99.2	−5.81

a G-factor – goodness factor and this value should be >−0.5 for a good model.

### Molecular dynamics simulations

**Figure 4:**
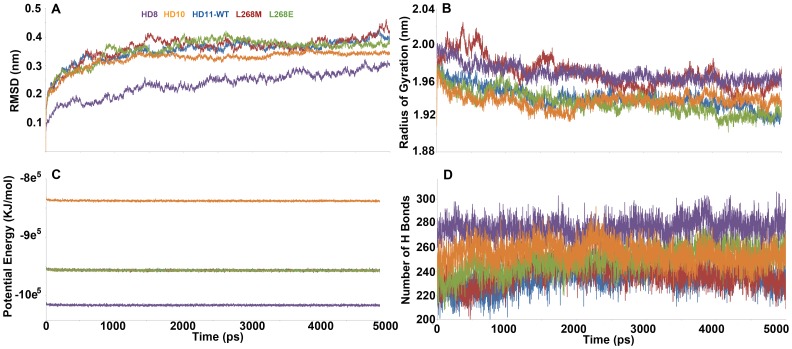
Basic MD analyses for all HDAC systems. (A) Cα-RMSD, (B) radius of gyration, (C) potential energy, and (D) number of hydrogen bonds of all HDAC systems.

Five systems including apoforms of HADC8, 10, 11-WT, 11-L268M and 11-L268E were subjected to 5 ns MD simulations. Our investigations started with the analyses to check the stability of all systems. Using tools available with GROMACS package, root mean square deviation (RMSD), root mean square fluctuation (RMSF), total number of intramolecular hydrogen bonds, energies, and radius of gyration were calculated to observe the stability of the systems. All systems considered were stable throughout the 5 ns MD simulations (Fig. 4). The HDAC8 structure was very stable compared to other systems as expected from its high structural stereochemical quality. All other systems were also stable throughout the simulation time. Other analyses based on radius of gyration (Rg), energies, and protein hydrogen bonds also have confirmed the stable nature of the systems during MD simulations (Fig. 4). The details of the size and environments of systems are listed in Table 3. The calculated average Cα RMSD values were 0.265, 0.338, 0.375, 0.381, and 0.380, respectively, for HDAC8, 10, 11-WT, 11-L268M, and 11-L268E systems (Fig. 4A). In terms of Rg calculation, interestingly, HDAC11 with a HDAC8 like mutation L268M has shown an Rg value similar to HDAC8 whereas HDAC11 WT and L268E were similar to HDAC10 (Fig. 4B). The potential energy and number of hydrogen bonds of all systems were stable during simulations (Fig. 4C and 4D). As another means of observing the stability of the systems the distance between His142 and Zn^2+^ ion, which are the important components of deacetylation machinery present in Zn-dependent HDAC enzymes was measured. In HDAC8 system the average distance between His142 and Zn was 0.469 Å whereas in HDAC10 and 11 systems the average distances were 0.724 Å and 0.656 Å, respectively. Interestingly, the mutated systems HDAC11-L268M and L268E have got the average distances of 0.433 and 0.468, respectively, close to that of HDAC8 system (Fig. S6 and Table 4). This result indicated that the single mutation introduced at the tunnel wall has brought significant changes in catalytic machinery and will influence the mechanism of action.

**Table 3:** System details of each model for MD simulations.

Model	Mutation	Number of water molecules	Number and type of counter ions
HDAC8	-	23243	12 Na^+^
HDAC10	-	25204	16 Na^+^
HDAC11	-	21943	2 Cl^−^
HDAC11	L268M	21944	2 Cl^−^
HDAC11	L268E	21944	1 Cl^−^

**Table 4:** The average distance values between important active site components in all isoforms measured during last 2 ns of the 5 ns long simulations.

Isoform	H*-Zn (Å)	1^st^ pair	2^nd^ pair	3^rd^ pair
HDAC8	H142-Zn	G151:M274	F208:Y306	F152:H180
	0.490	1.456	0.978	0.791
HDAC10	H134-Zn	G143:E272	F205:Y305	F144:H174
	0.680	1.904	1.813	1.340
HDAC11-WT	H142-Zn	G151:L268	Y209:Y304	F152:H183
	0.703	0.990	0.730	0.526
HDAC11-L268M	H142-Zn	G151:M268	Y209:Y304	F152:H183
	0.444	1.025	0.700	0.788
HDAC11-L268E	H142-Zn	G151:E268	Y209:Y304	F152:H183
	0.470	0.944	0.982	0.641

* H-histidine.

### Validation of MD simulation results

In first validation procedure, a set of co-crystallized HDAC8 inhibitors were selected and docked into the active site of the HDAC8 structure subjected to MD simulation. All of the docked inhibitor conformations were similar to the conformation observed in the crystal structure and did not lose the important interactions including the metal ion contact (Figure S7). In the second validation procedure, a set of known HDAC8, 10 and 11 inhibitors (32, 20 and 14 compounds respectively) were collected from the literature with their inhibitory profiles (IC_50_ values). These known inhibitors were docked and a correlation was observed between their inhibitory profiles and docking scores. The results obtained from this validation procedure also confirmed that the structures obtained from MD simulations are reliable to be used in further analyses (Table S1).

### Distances between tunnel forming residues

**Figure 5:**
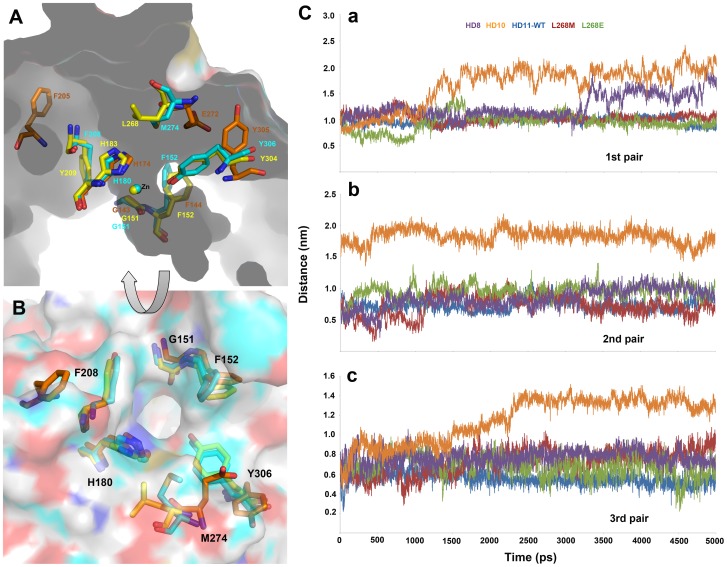
Comparison of distances between tunnel forming residue pairs in all HDACs. (A) Inner view of the tunnel in HDAC8 and conserved tunnel forming residues present in HDAC8, 10 and 11 enzymes. (B) Top view of the tunnel and the residues arrangement. (C) The distances calculated between three pairs of tunnel forming residues in all systems.

Amino acid residues that are reported to form tunnel of the active site of HDAC enzymes were identified in different isoforms (HDAC10 and 11) based on the crystal structure information available for HDAC8 of class I. The homology models of HDAC10 and 11 were also overlaid on the structure of HDAC8 to compare the size of the tunnels formed in these three different HDAC isoforms. The first of three pairs of the tunnel forming residues is G151:M274 in HDAC8. The equivalent residues of this position in HDAC10 and 11 were G143:E272 and G151:L268, respectively. The glycine that is part of the first pair is conserved in all isoforms but in the other side it is different (M in HDAC8, E in HDAC10, and L in HDAC11). The second pair of tunnel forming residues is F208:Y306, F205:Y305, and Y209:Y304 in HDAC8, 10, and 11, respectively. These second pair of residues was very much conserved in all isoforms except for Y209 in HDAC11. Third pair of residues including F152:H180, F144:H174, and F152:H183 in HDAC8, 10, and 11, respectively, was completely conserved in all isoforms. The distances between these residues were calculated to observe the changes in the size of the tunnel during simulation. Comparison of initial tunnels between these three isoforms has shown that the tunnels of HDAC8 and HDAC11 were narrow and deep to the bottom where the catalytic machinery containing divalent Zn ion and charge relay system is present. The initial tunnel observed in HDAC10 was very wide and shallow compared to that of HDAC8 and 11. This could be because of the long distance between the second pair (F205:Y305) of tunnel forming residues and except this pair of residues all other amino acids were at similar locations as in HDAC8 and HDAC11 (Fig. 5A). The distances between these residues were observed throughout the simulation time to investigate their stability. The distances between the first pair of residues of all systems were calculated for last 3 ns of simulations and compared. The result of this comparison revealed that the distance between first pair in HDAC8 was very stable (1.112 nm) during first half of the simulation time and became wider (1.456 nm) in the second half. The distance between first pair in HDAC10 has become wide after 1 ns of simulation time and this was observed because of the fluctuation of E272, which is the only acidic and less hydrophobic residue equivalent to other isoforms (M and L in HDAC8 and 11, respectively). In case of HDAC11 and its mutated forms, the distances were maintained very stable throughout the simulation time (Fig. 5Ca and Table 4). The calculated distances between second pair of residues in all systems were stable during the simulation but interestingly difference was observed between HDAC11 and its mutational systems. Particularly, the distance in HDAC10-like mutation system (L268E) has increased (0.982 nm) compared to HDAC11 WT (0.730) and HDAC8-like mutation (L268M, 0.700 nm) which were very similar to each other. The second pair distance was too large in HDAC10 system (1.813 nm) when compared to any other systems as this is the nature of the tunnels present in class IIb HDACs. This is confirmed by comparing the crystal structures of other class IIb HDACs like HDAC4 and 2. The distance observed in HDAC8 system for second pair of residues (0.978 nm) was similar to HDAC11 systems. These analyses of distances between second pair of residues in HDAC11 and its mutation systems revealed that L268E mutation has brought a significant change in tunnel size (Fig. 5Cb). The distances between third pair (F152:H180) of residues forming tunnel in HDAC8, 10, and 11 isoforms were calculated. The results revealed that the distance in HDAC10 system has highly deviated after 1.5 ns of simulation time (Fig. 5Cc). This distance in HDAC10 has become wide in the later part of simulation showing 1.340 nm as average distance between third pair of residues. This distance in HDAC8 (0.791 nm) was completely stable throughout the simulation. In terms of HDAC11 and its mutation systems, L268M has shown wider average distance (0.788 nm) when compared to the WT (0.526 nm) and L268E (0.641 nm) counterparts (Fig. 5Cc).

### Tunnel size comparison in HDAC8, 10, and 11 isoforms

**Figure 6:**
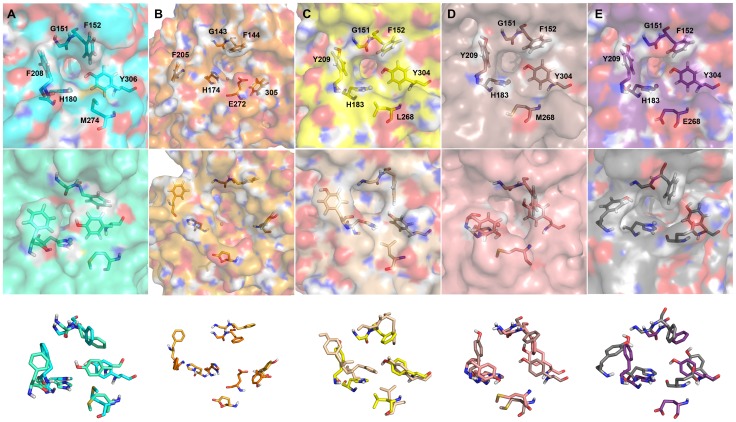
Surface views showing tunnels and tunnel forming residues of initial and average structures. (A) Initial and average structures of HDAC8 are shown in cyan and pale green colors. (B) Initial and average structures of HDAC10 are shown in dark and light orange colors. (C) Initial and average structures of HDAC11 are shown in yellow and wheat colors. (D) Initial and average structures of HDAC11 L268M are shown in dark salmon and salmon colors. (E) Initial and average structures of HDAC11 L268E are shown in violet and grey colors, respectively.

**Figure 7:**
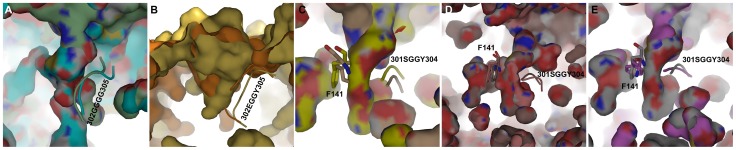
The arrangement of tunnel-lining residues in initial and average structures of all HDAC systems. (A) Initial and average structures of HDAC8 are shown in cyan and pale green colors. (B) Initial and average structures of HDAC10 are shown in dark and light orange colors. (C) Initial and average structures of HDAC11 WT are shown in yellow and wheat colors. (D) Initial and average structures of HDAC11 L268M are shown in dark salmon and salmon colors. (E) Initial and average structures of HDAC11 L268E are shown in violet and grey colors, respectively.

The overall effect of movement of tunnel forming residues was analyzed by investigating the size of the tunnels by various means. The partially transparent surface representation of the initial and average structure of proteins enabled us comparing not only the shape of the tunnel but also the visualization of location of tunnel forming residues. The tunnel present in the initial structure of HDAC8 is very narrow and deep with an approximate width of 3.674 Å and a volume of 54.25 Å^3^. This tunnel has become further narrow during MD simulation because of the movement of the tunnel forming residues (Fig. 6A). The width and volume of the tunnel present in the average structure of the MD simulation were 3.202 Å and 51.50 Å^3^, respectively. A set of amino acids forming a variable region that lines the sidewall of the tunnel in HDAC was identified and the movement of this region influences the size of the tunnels. Four consecutive glycine residues (G302–G305) form this region in HDAC8 isoform and the movement of this region has narrowed down the size of the tunnel in HDAC8 system (Fig. 7A). The tunnel present in HDAC10 isoform was wide compared to other systems and it became wider during the simulation because of the movement of E272 away from its tunnel forming pair G143 (Fig. 6B). At the mean time the tunnel has become deeper due to the movement of tunnel-lining region formed by E302, G303, G304 and Y305 (E302GGY305) amino acid residues that are equivalent to the HDAC8 tunnel-lining region. This tunnel-lining region has moved away from its initial position during MD simulation and making the tunnel deep and increasing the volume of the tunnel as well (Fig. 7B). Interestingly, during the simulation of HDAC11 and its mutation systems we have observed various changes at the tunnels. The initially formed HDAC8-like narrow and deep tunnel has become wide and less deep during the simulation time. This change was due to the slight movement of all three pairs of tunnel forming residues (Fig. 6C). The equivalent tunnel-lining residues present in HDAC11 are S301, G302, G303, and Y304 along with another aromatic amino acid F141 that is present at the other side of the tunnel. These lining residues determined the size of the tunnel in HDAC11 and its mutation systems. In HDAC11 WT system, the initial deep tunnel was blocked at half the way by the movement of F141 and tunnel-lining residues (Fig. 7C). The shape of the tunnel in HDAC11 L268M system has become slightly wide compared to that of the initial tunnel. This change has been brought to the structure due to the movement of second and third pairs of tunnel forming residues (Fig. 6D). Unlike in the HDAC11 WT system the tunnel-lining region and F141 have moved away from each other forming a wide bottom and did not affected the depth of the tunnel in HDAC11 L268M system (7D). The tunnel present in HDAC11 L268E system has become very wide at sides mainly by the movement of the mutated (L268E) residue and Y209 (Fig. 6E). Interestingly, the movement of these residues in this system has resembled the result observed in HDAC10 system. The surface representation of the tunnel region also has shown a tunnel similar to that of HDAC10 (Fig. 6B and 6E). The movements of tunnel-lining residues and F141 at bottom of the tunnel have blocked the deep tunnel half the way and brought a shallow and wide bottom as observed in HDAC10 (Fig. 7E). A single mutation at the tunnel forming residues has brought substantial changes in the size of the tunnel and thus giving clues on the substrate and inhibitor specificity of every HDAC isoforms, which has been a challenge to the researchers till date.

### Overall structural changes in the systems

**Figure 8:**
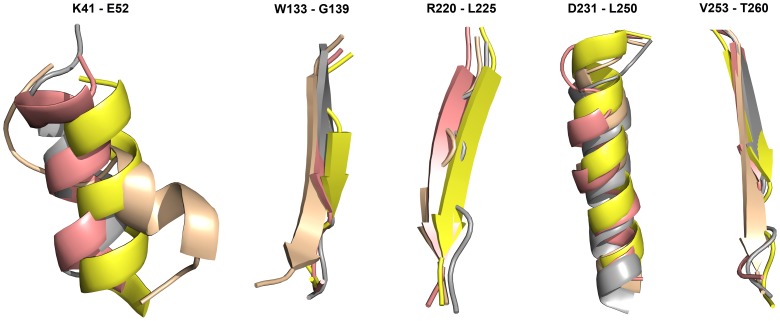
Secondary structural changes observed in HDAC11 WT and mutational systems. Initial and MD average structure of HDAC11 WT is shown in yellow and wheat color. L268M and L268E systems are shown in salmon and grey color.

Comparison of initial and representative structures of every system has shown major structural changes that were brought during the time of simulation. HDAC8 system was very stable during simulation and thus not major secondary structural changes were observed except an elongation of a beta sheet formed by A259 to G265 residues and a slight unfolding of a helix (P22 to S30) (Fig. S8A). In HDAC10 system, number of secondary structure changes was observed such as unfolding of helices and shortening and elongation of beta sheets (Fig. S8B). The beta sheet of HDAC10 formed by L5 to E9 was elongated and another beta sheet (C47 to L50) was formed newly during the simulation. Two other beta sheets formed by V189-H195 and V223-W228 residues were shortened and disappeared, respectively, while helices formed by P279-V291 and N235-F253 residues were slightly unfolded. In terms of HDAC11 WT system the helix formed by K41-E52 residues was almost completely unfolded and the three amino acids (A134-N136) long beta sheet was extended to a seven residues (W133-G139) long beta sheet during the simulation. Addition to these changes, another five residues long beta sheet (V253-N257) has become eight residues longer (V253-T260) (Fig. 8). In both mutation systems the unfolded helix was stable unlike WT system and the three residues (A134-N136) long beta sheet was elongated as observed in WT system. In addition, in mutation systems the long helix formed by residues between D231 and L250, which was unchanged in WT system, was partially unfolded during the simulation. In particular, the beta sheet formed by R220-L225 residues of HDAC11 system completely disappeared in HDAC11-L268E as exactly observed in HDAC10 (V223-W228). This also indicated that L268E system behaved similar to HDAC10 system. These major secondary structural changes observed in different HDAC systems have brought the differences at the tunnel. The changes observed in variable regions are not discussed in this study.

## Conclusions

The MD simulations of apoforms of three different HDAC isoforms, namely, HDAC8, 10 and 11 from class I, II and IV, respectively, were performed for 5 ns using GROMACS simulation package. Homology models of HDAC10 and 11 were built using comparative modeling procedure and validated for their stereochemical quality. These models and HDAC8 were subjected to MD simulations to study the tunnel-like active site and its behavior. The main aim of this study is to find a valid reason for the observed substrate or inhibitor specificity among HDAC isoforms. The comparison of the results of the MD simulations of these systems revealed that there are subtle differences among HDAC isoforms that can be used in designing isoform specific inhibitors as novel anti-cancer therapeutics. In our study, we have identified a particular amino acid, not conserved in HDAC isoforms, present in the side walls of the tunnel-like active site. The importance of this particular residue in different isoforms has been investigated by mutating it to M and E as present in other isoforms. These studies have disclosed various information including the importance and influence of these residues in maintaining the integrity of the tunnel in respective isoform. Moreover, the results of this study have also proved that HDAC11 is phylogenetically close to HDAC8 than HDAC10 as it has shown similar behavior during MD simulations. At the same time a single HDAC10-like mutation has brought HDAC10-like changes in HDAC11 system. The tunnel-lining residues present at the bottom of the tunnel also are very much conserved in all three systems and had influenced in maintaining the size of the tunnels. The results of this study can successfully be utilized in future designing of isoform specific potential HDAC inhibitors. Very few studies were performed on HDAC11 as not enough structural information is available. This study has developed a good model for this isoform which can give reliable structural information for receptor-based drug design.

## Supporting Information

Figure S1 **Comparison of tunnel like active sites.** Surface views of (A) HDAC8, 4 and 7 enzyme crystal structures (PDB codes 1T69, 2VQJ and 3C10, respectively) with calculated electrostatic potentials showing different size of tunnels.
(TIF)

Figure S2 **Sequence alignment between HDAC8 and the template.** This was used in building missing HDAC8 regions.
(TIF)

Figure S3 **Sequence alignment between HDAC10 and the template HDAC4 enzyme.**
(TIF)

Figure S4 **Sequence alignment between HDAC11 and its template HDAC8.**
(TIF)

Figure S5 **Structure validation of the HDAC homology models.** Ramachandran plots of (A) HDAC8, (B) HDAC10, and (C) HDAC11. (D) PROSA result for all HDAC models. HDAC8 and 11 models are shown in black and red colors. HDAC10 models based on one and two templates are shown in green and blue colors, respectively.
(TIF)

Figure S6 **The distances between His142 and Zn ion present in all systems.**
(TIF)

Figure S7 **Molecular docking results to validate the structures obtained from MD simulations.** Four crystal structure conformations of three different inhibitors at the active sit of HDAC8 were compared with the molecular docking results. (A) co-crystalized SAHA (PDB code: 1T69) is in blue and the docked conformation in pale green color, (B) co-crystalized V5X (PDB code: 2V5X) is in white and the docked conformation in dark salmon color, (C) co-crystalized TSA (PDB code: 1T64) is in violet and the docked conformation in yellow color, (D) co-crystalized TSA (PDB code: 3F0R) is in lime and the docked conformation in yellow color.
(TIF)

Figure S8 **Secondary structural changes observed in HDAC8 and 10 systems.** (A) Initial and average structures of HDAC8 are shown in cyan and pale green colors, respectively. (B) Initial and average structures of HDAC10 are shown in dark and light orange colors, respectively.
(TIF)

Table S1 The sets of inhibitors for HDAC8, 10 and 11 isoforms (32, 20 and 14 compounds respectively) used in molecular docking validation. The inhibitory profiles, GOLD fitness scores, and the correlation between them for individual HDAC isoforms are provided.
(DOCX)

Text S1
