# Supplementary figures and images for: Molecular Modeling Study on Tunnel Behavior in Different Histone Deacetylase Isoforms
Source: PLoS One. 2012 Nov 29;7(11):e49327. doi: 10.1371/journal.pone.0049327 (PMC3510210; doi:10.1371/journal.pone.0049327)

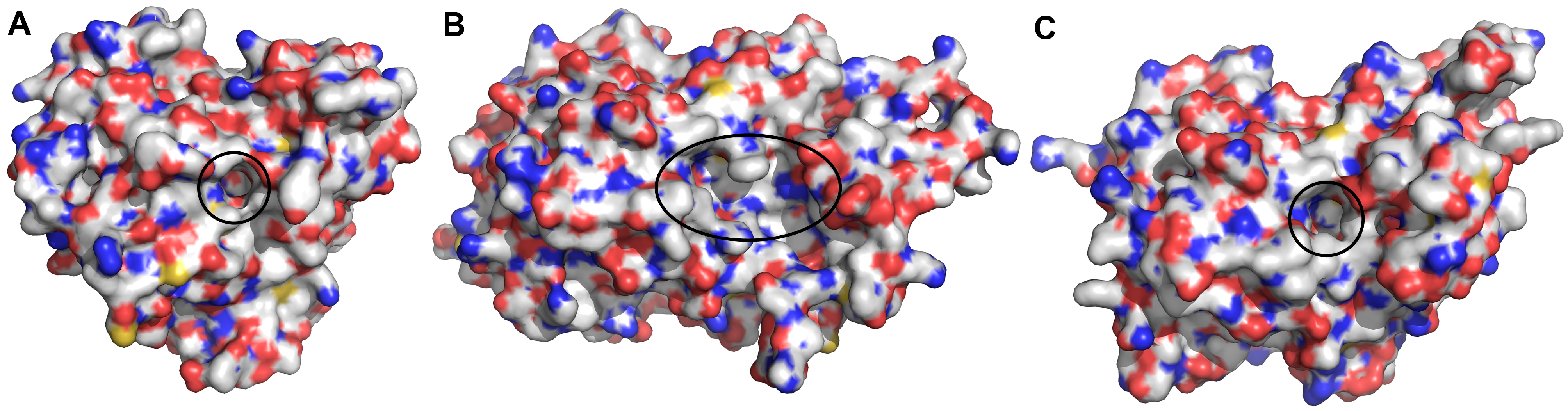

Supplement: Figure S1 — Comparison of tunnel like active sites. Surface views of (A) HDAC8, 4 and 7 enzyme crystal structures (PDB codes 1T69, 2VQJ and 3C10, respectively) with calculated electrostatic potentials showing different size of tunnels. (TIF) [file pone.0049327.s001.tif]

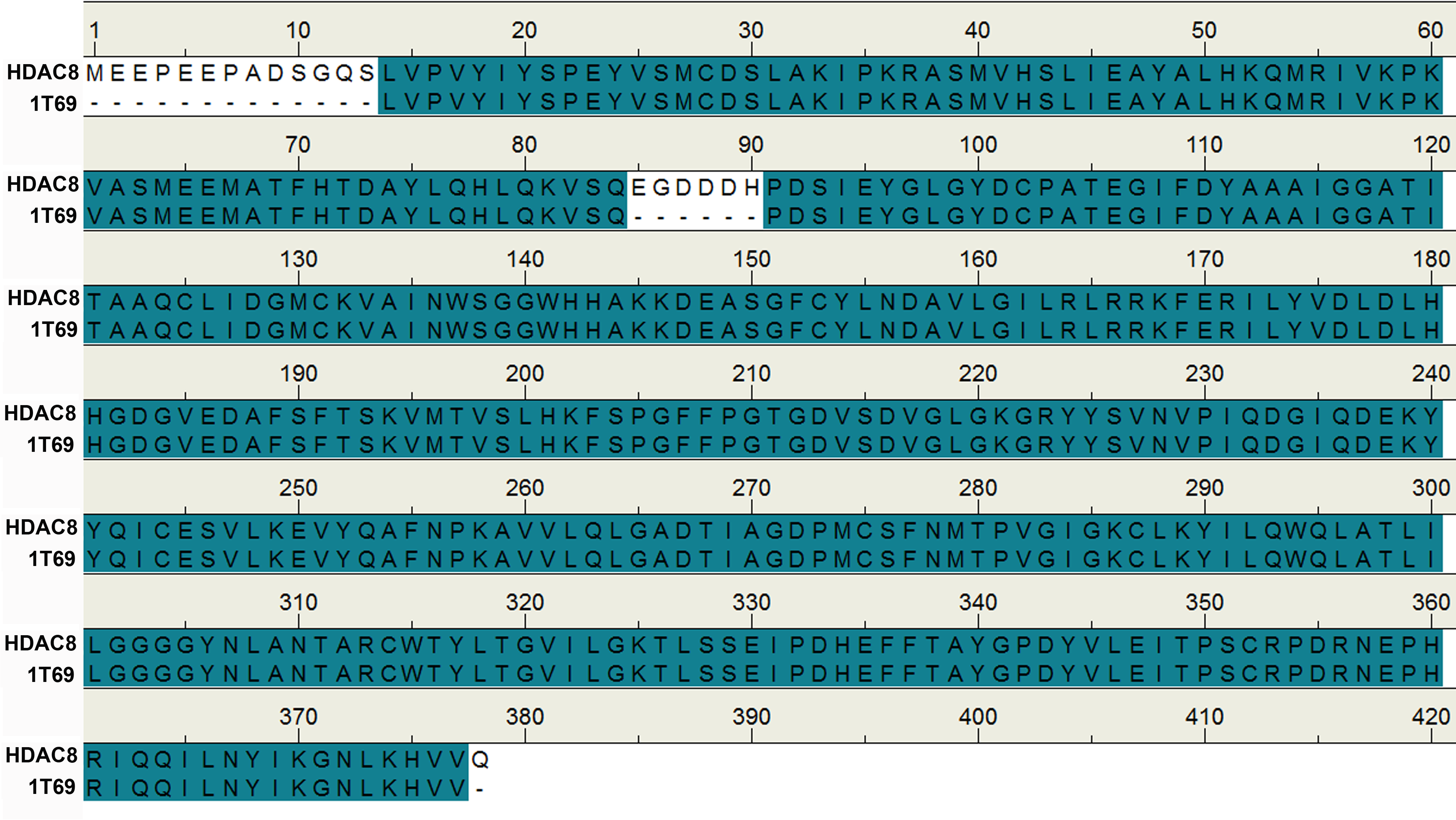

Supplement: Figure S2 — Sequence alignment between HDAC8 and the template. This was used in building missing HDAC8 regions. (TIF) [file pone.0049327.s002.tif]

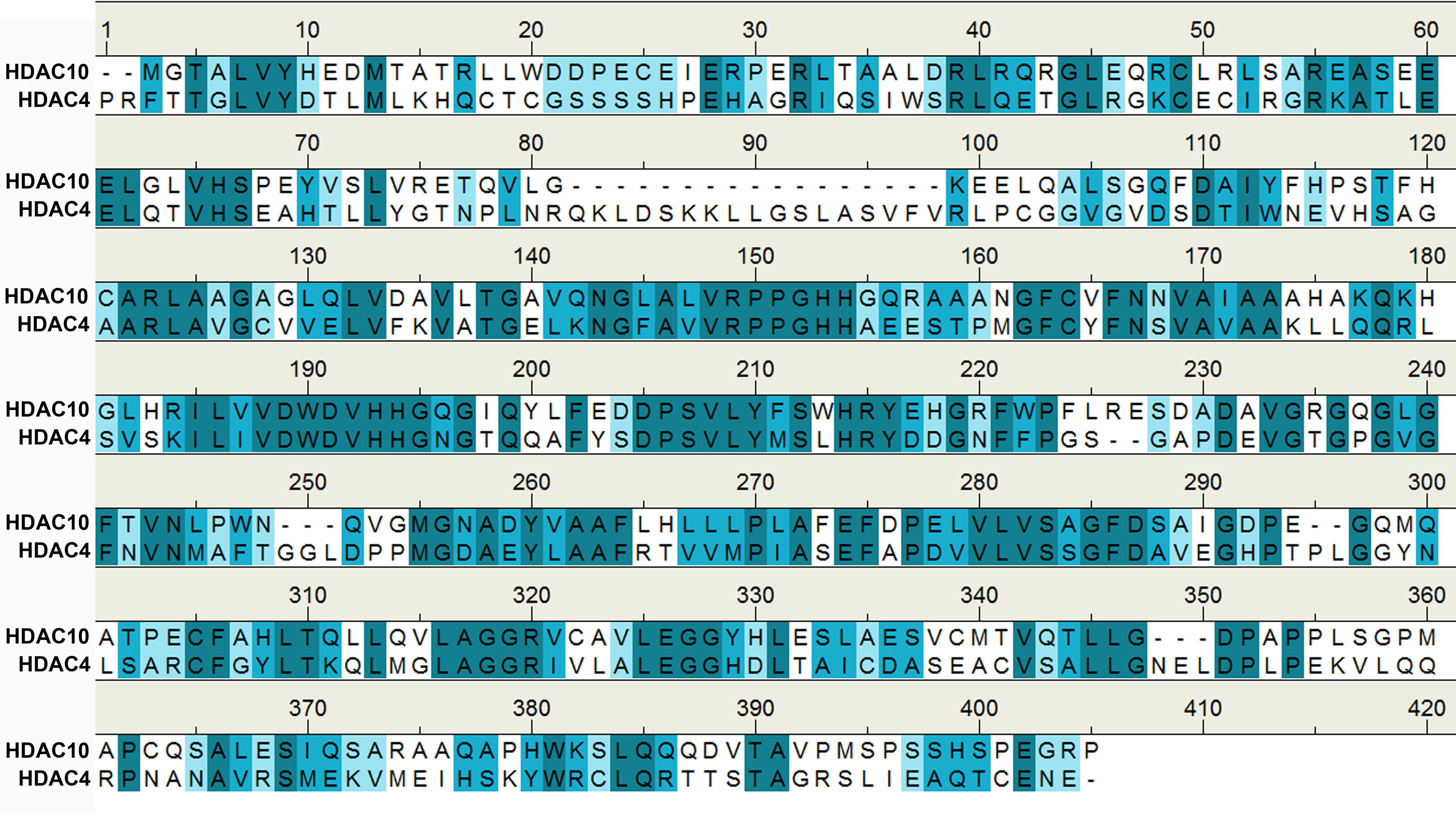

Supplement: Figure S3 — Sequence alignment between HDAC10 and the template HDAC4 enzyme. (TIF) [file pone.0049327.s003.tif]

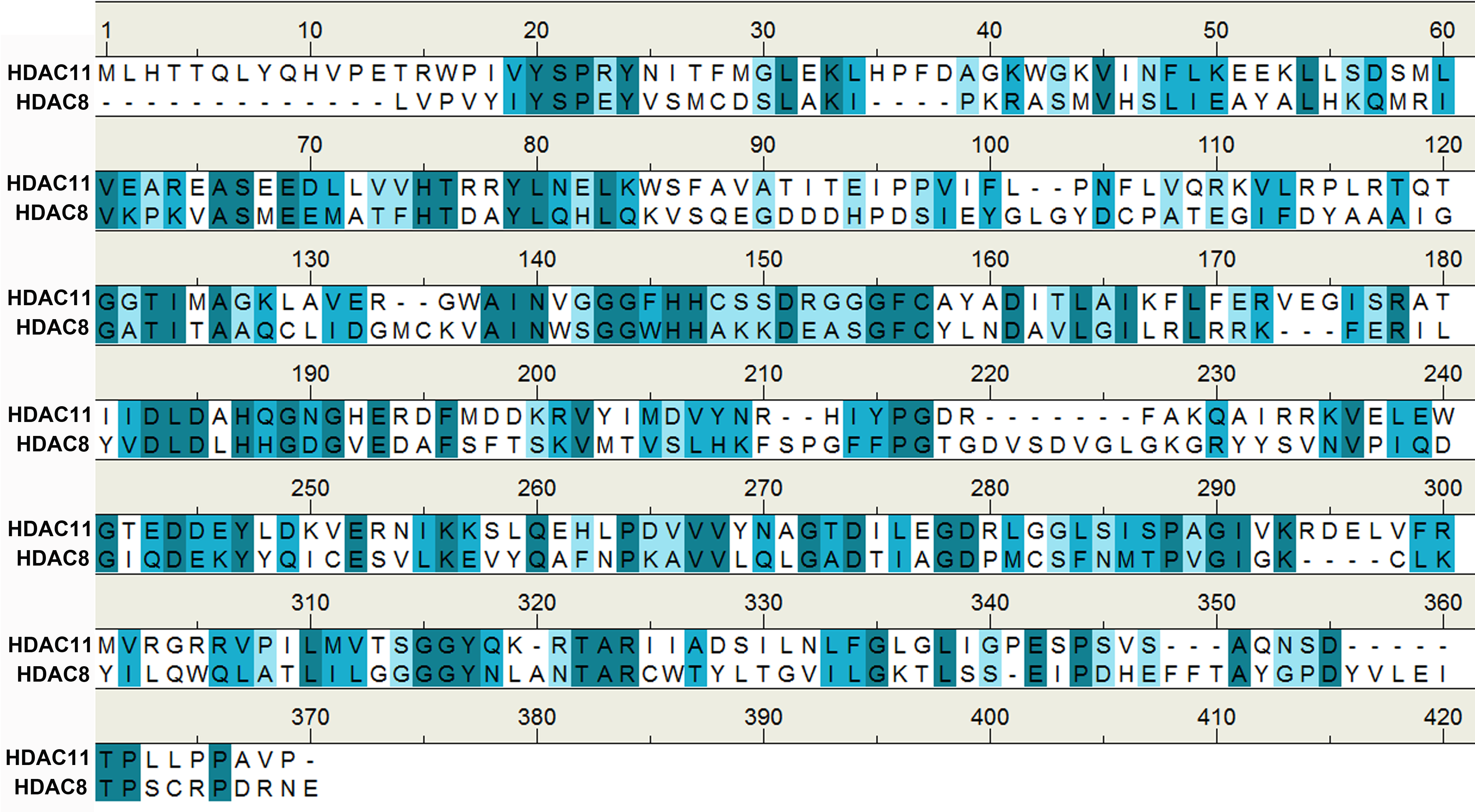

Supplement: Figure S4 — Sequence alignment between HDAC11 and its template HDAC8. (TIF) [file pone.0049327.s004.tif]

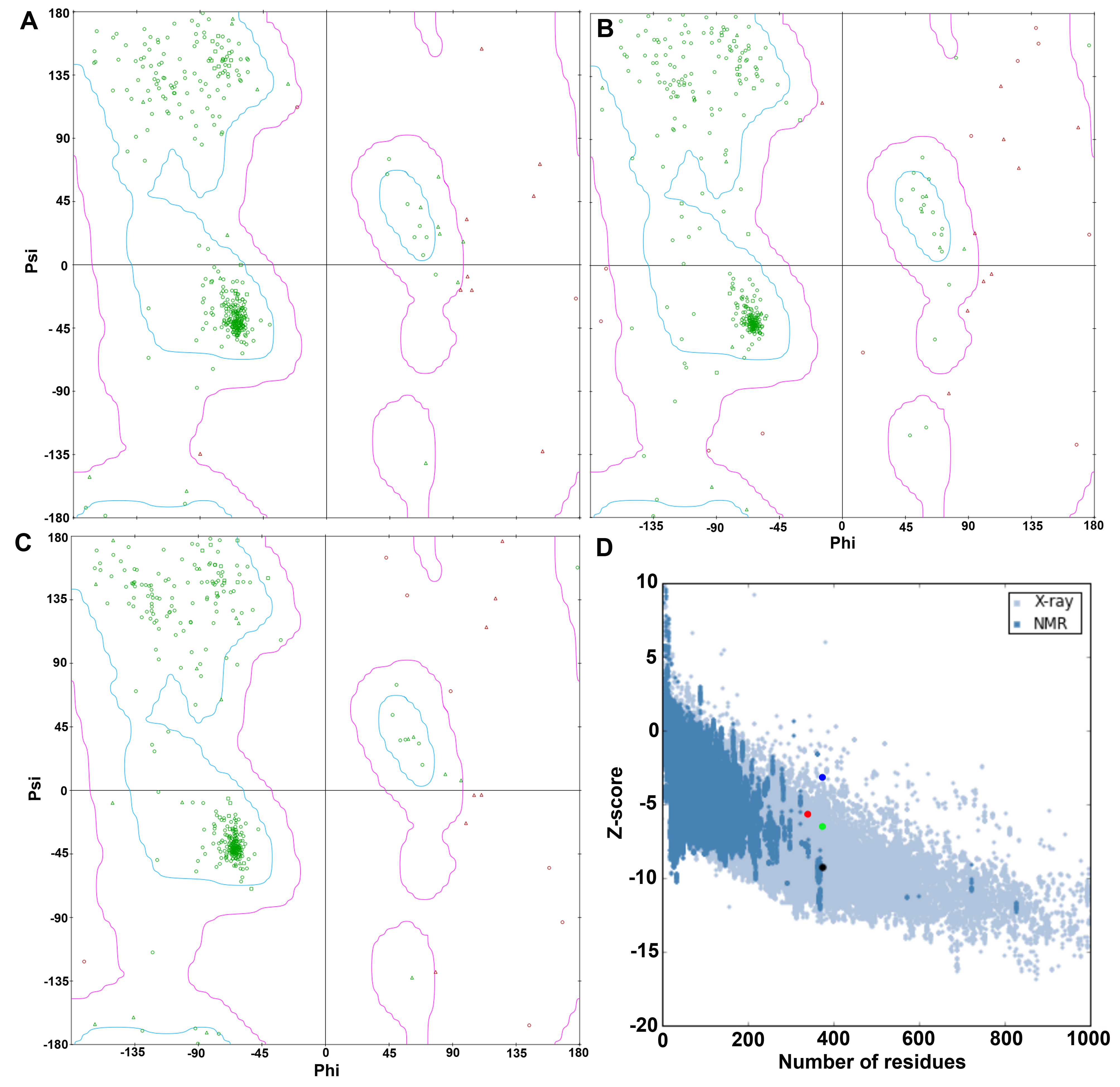

Supplement: Figure S5 — Structure validation of the HDAC homology models. Ramachandran plots of (A) HDAC8, (B) HDAC10, and (C) HDAC11. (D) PROSA result for all HDAC models. HDAC8 and 11 models are shown in black and red colors. HDAC10 models based on one and two templates are shown in green and blue colors, respectively. (TIF) [file pone.0049327.s005.tif]

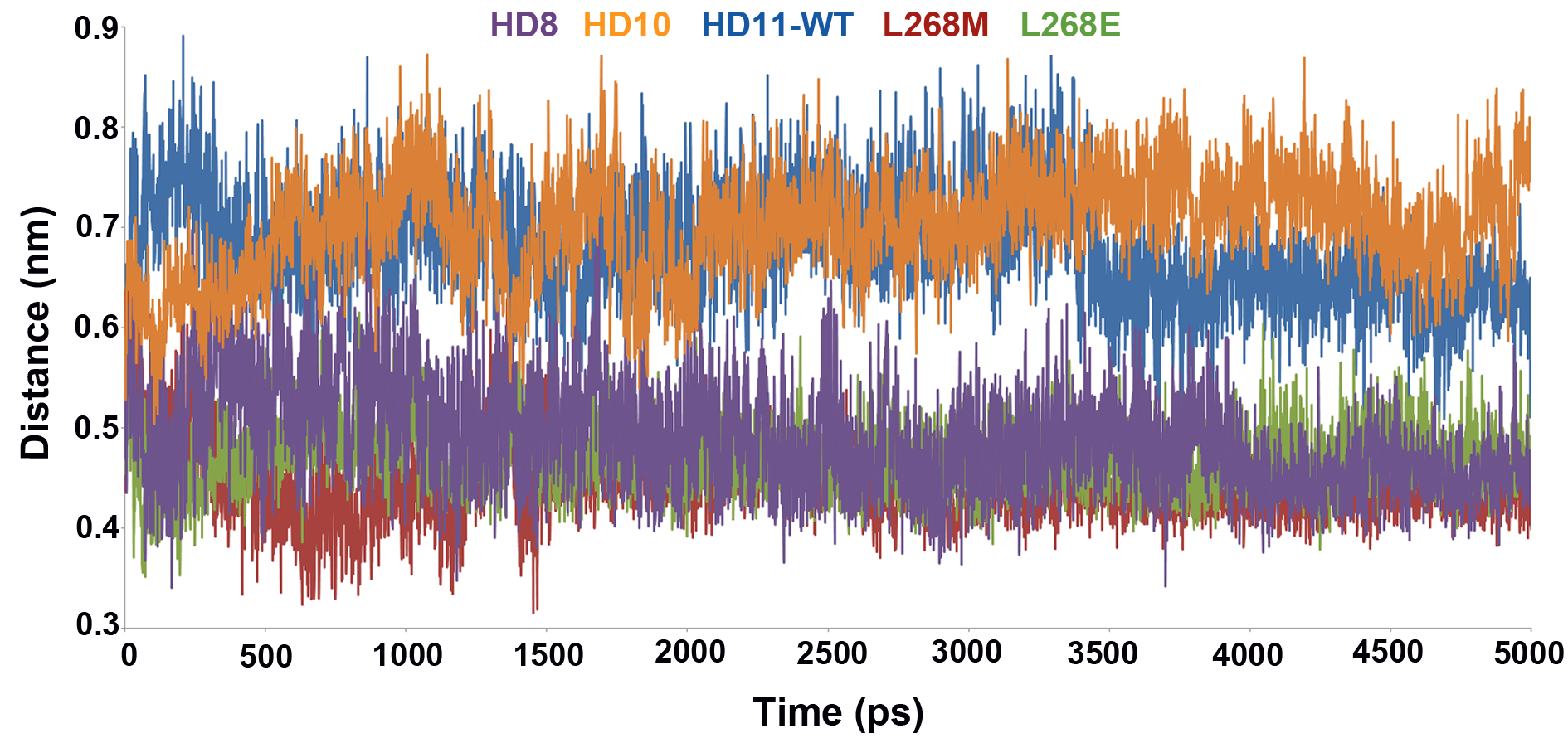

Supplement: Figure S6 — The distances between His142 and Zn ion present in all systems. (TIF) [file pone.0049327.s006.tif]

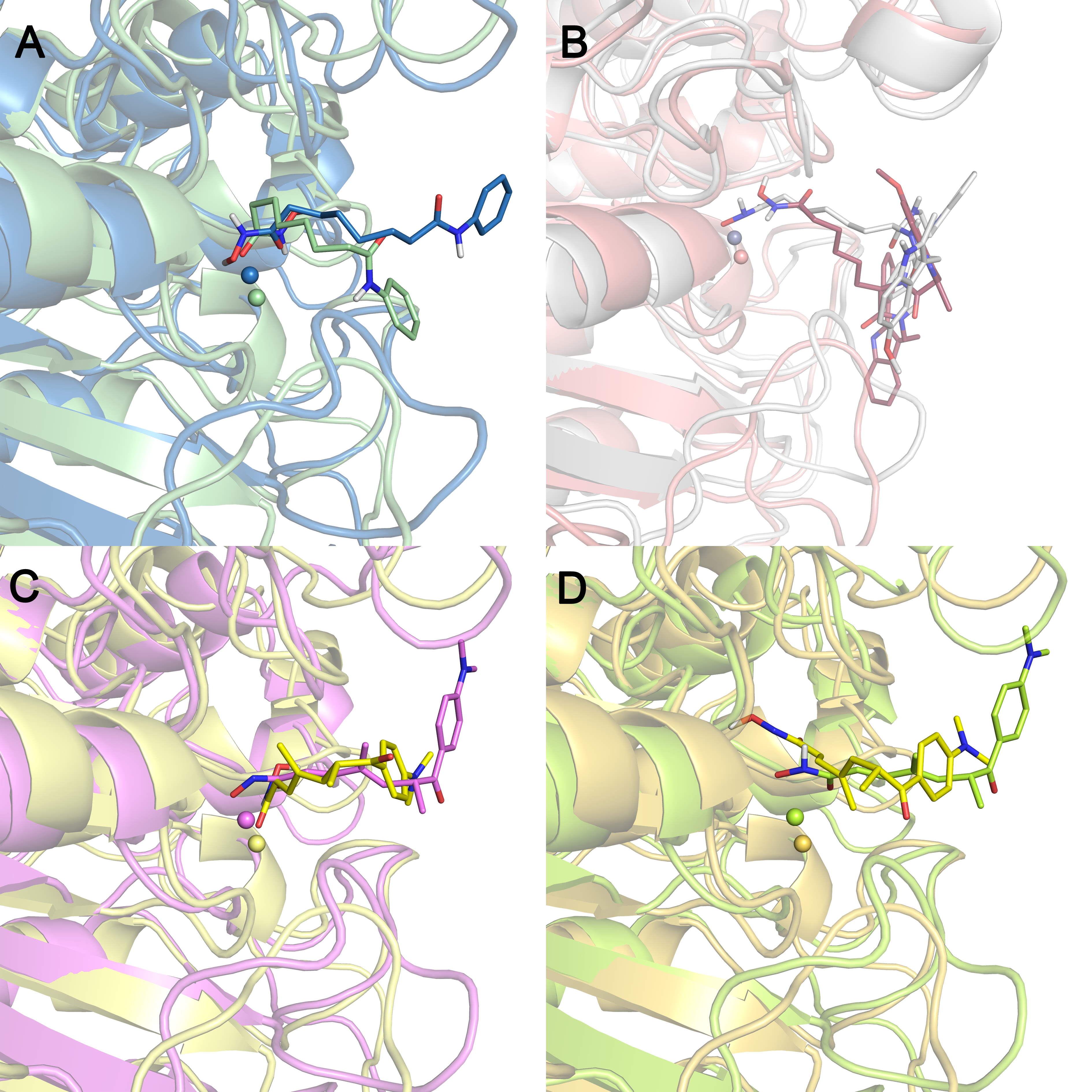

Supplement: Figure S7 — Molecular docking results to validate the structures obtained from MD simulations. Four crystal structure conformations of three different inhibitors at the active sit of HDAC8 were compared with the molecular docking results. (A) co-crystalized SAHA (PDB code: 1T69) is in blue and the docked conformation in pale green color, (B) co-crystalized V5X (PDB code: 2V5X) is in white and the docked conformation in dark salmon color, (C) co-crystalized TSA (PDB code: 1T64) is in violet and the docked conformation in yellow color, (D) co-crystalized TSA (PDB code: 3F0R) is in lime and the docked conformation in yellow color. (TIF) [file pone.0049327.s007.tif]

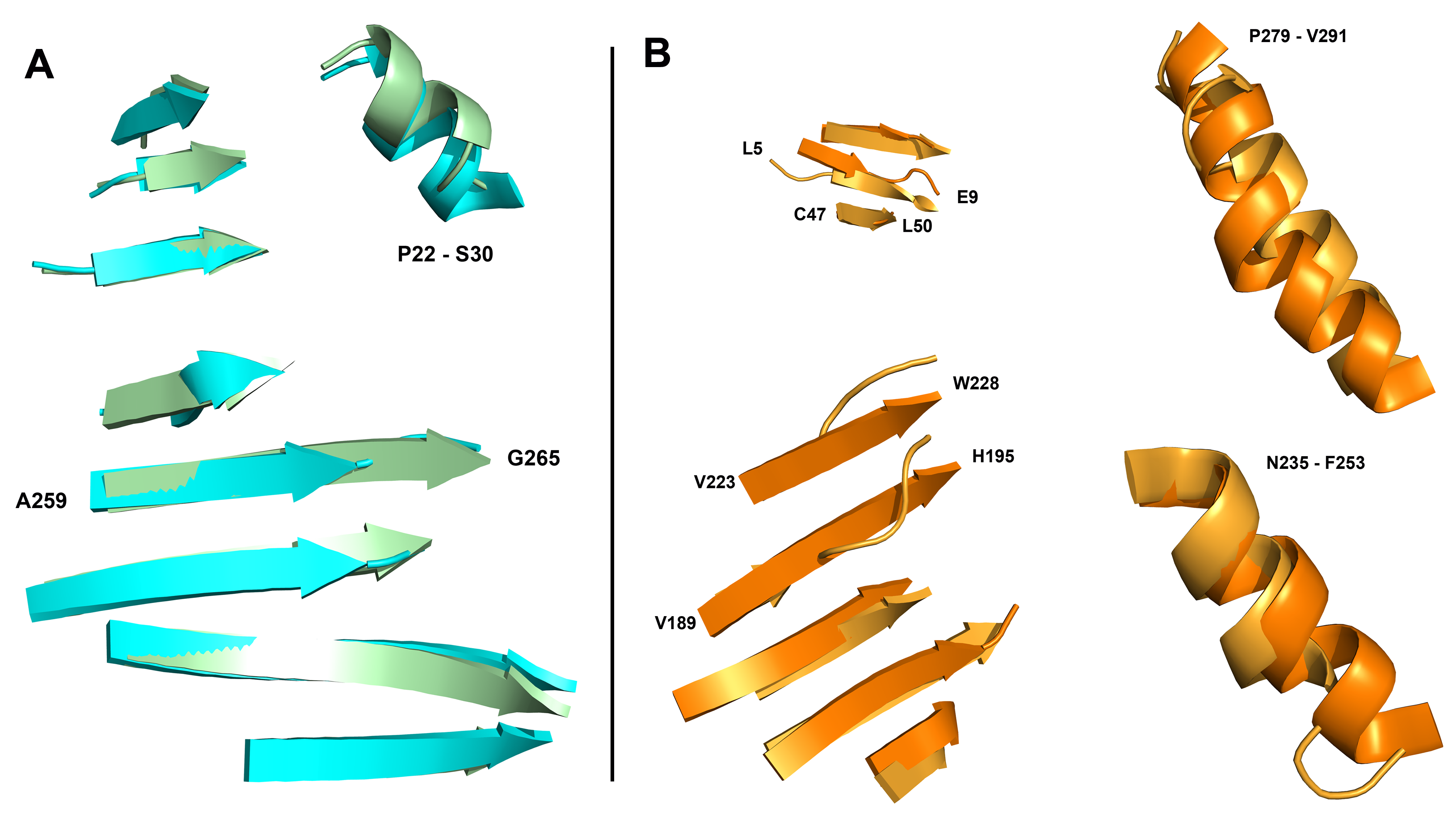

Supplement: Figure S8 — Secondary structural changes observed in HDAC8 and 10 systems. (A) Initial and average structures of HDAC8 are shown in cyan and pale green colors, respectively. (B) Initial and average structures of HDAC10 are shown in dark and light orange colors, respectively. (TIF) [file pone.0049327.s008.tif]
