# Supplementary material for: Molecular Modeling Study on Tunnel Behavior in Different Histone Deacetylase Isoforms
Source: PLoS One. 2012 Nov 29;7(11):e49327. doi: 10.1371/journal.pone.0049327 (PMC3510210; doi:10.1371/journal.pone.0049327)
Supplement: Table S1 — The sets of inhibitors for HDAC8, 10 and 11 isoforms (32, 20 and 14 compounds respectively) used in molecular docking validation. The inhibitory profiles, GOLD fitness scores, and the correlation between them for individual HDAC isoforms are provided. (DOCX) [file pone.0049327.s009.docx]

**Table S1.** The sets of inhibitors for HDAC8, 10 and 11 isoforms (32, 20 and 14 compounds respectively) used in molecular docking validation. The inhibitory profiles, GOLD fitness scores, and the correlation between them for individual HDAC isoforms are provided.

| **Serial No.** | **HDAC8** | | **HDAC10** | | **HDAC11** | |
| --- | --- | --- | --- | --- | --- | --- |
|  | **IC_50_** | **Dock Score** | **IC_50_** | **Dock Score** | **IC_50_** | **Dock Score** |
| 1 | 17 | 68.48 | 4.3 | 73.1673 | 5.58 | 56.3601 |
| 2 | 20 | 69.11 | 10.7 | 70.3542 | 13.9 | 55.9358 |
| 3 | 31 | 67.41 | 11.1 | 71.3621 | 22.9 | 55.889 |
| 4 | 50 | 68.48 | 14.5 | 71.6004 | 100 | 56.5412 |
| 5 | 63 | 74.80 | 44.9 | 70.0947 | 103 | 63.8928 |
| 6 | 63 | 69.71 | 73 | 68.3143 | 174 | 58.1499 |
| 7 | 79 | 69.18 | 176 | 63.3656 | 546 | 60.9542 |
| 8 | 79.8 | 67.56 | 178 | 62.8012 | 564 | 56.3274 |
| 9 | 86 | 73.85 | 190 | 67.2331 | 3,060 | 54.9101 |
| 10 | 87 | 72.44 | 200 | 65.8218 | 5,480 | 58.4352 |
| 11 | 90 | 69.96 | 240 | 69.7924 | 6,260 | 59.6223 |
| 12 | 120 | 70.08 | 250 | 62.9545 | 10,800 | 63.747 |
| 13 | 139 | 67.87 | 254 | 66.2778 | 16,800 | 62.6245 |
| 14 | 140 | 72.96 | 852 | 63.1818 | 34,100 | 67.4653 |
| 15 | 151 | 66.63 | 1,530 | 60.8504 |  |  |
| 16 | 197 | 70.04 | 1,580 | 59.4505 |  |  |
| 17 | 328 | 70.45 | 4,700 | 58.9396 |  |  |
| 18 | 487 | 68.13 | 6,100 | 59.0453 |  |  |
| 19 | 553.45 | 68.88 | 7,360 | 58.9126 |  |  |
| 20 | 690 | 69.39 | 7,840 | 57.3168 |  |  |
| 21 | 690 | 68.41 |  |  |  |  |
| 22 | 759 | 69.32 |  |  |  |  |
| 23 | 922 | 68.64 |  |  |  |  |
| 24 | 980 | 67.19 |  |  |  |  |
| 25 | 1,280 | 64.19 |  |  |  |  |
| 26 | 1,310 | 65.46 |  |  |  |  |
| 27 | 1,900 | 64.26 |  |  |  |  |
| 28 | 1,970 | 62.90 |  |  |  |  |
| 29 | 2,010 | 66.06 |  |  |  |  |
| 30 | 2,710 | 63.02 |  |  |  |  |
| 31 | 4,120 | 62.71 |  |  |  |  |
| 32 | 5,220 | 62.57 |  |  |  |  |
|  | Correlation | 0.567 | Correlation | 0.555 | Correlation | 0.568 |
