# Supplementary material for: Molecular Modeling Study on Tunnel Behavior in Different Histone Deacetylase Isoforms
Source: PLoS One. 2012 Nov 29;7(11):e49327. doi: 10.1371/journal.pone.0049327 (PMC3510210; doi:10.1371/journal.pone.0049327)
Supplement: Text S1 [file pone.0049327.s010.docx]

**Text S1:**

In building homology model for HDAC10, the closest homolog identified by BLAST tool was the crystal structure of catalytic domain of HDAC4 bound with trifluoromethyl ketone inhibitor (PBD code: 2VQJ). Sequence alignment between HDAC10 and HDAC4 has shown 34.9% and 53.5% of identity and similarity, respectively (Fig. S3). In terms of building homology model of HDAC11, the crystal structure of HDAC8 (PDB code: 1T64), the closest homolog identified by BLAST tool, was used as template. Though 1T64 was found to have unusual binding of the inhibitor, TSA, with two adjacent binding pockets the resulted homology model of HDAC11 was not built with two binding pockets but one as any other HDAC isoforms. The sequence alignment between the primary sequences of HDAC11 and HDAC8 enzymes had shown the identity and similarity of 22.2% and 39.6%, respectively (Fig. S4).
